# Consumers’ Perceptions of the Australian Health Star Rating Labelling Scheme

**DOI:** 10.3390/nu12030704

**Published:** 2020-03-06

**Authors:** Fiona E. Pelly, Libby Swanepoel, Joseph Rinella, Sheri Cooper

**Affiliations:** 1 University of the Sunshine Coast, Sunshine Coast, QLD 4558, Australia; lswanepo@usc.edu.au (L.S.); joseph.rinella@gmail.com (J.R.); 2 Southern Cross University, Gold Coast, QLD 4558, Australia; sheri.cooper@scu.edu.au

**Keywords:** front-of-pack labelling, health star rating, nutrition labelling, consumer perception, qualitative research

## Abstract

The objective of this study was to explore consumers’ use and perception of the Australian Health Star Rating (HSR). A purposive sample of fifteen Australian grocery shoppers was recruited into four focus groups using a supermarket intercept strategy. Focus group discussions were recorded, transcribed and analysed using an iterative approach to thematic analysis. Three key themes emerged from analysis. The HSR was seen as simple, uncluttered, easy to understand and useful for quick comparison across products. The nutrition information was viewed positively; however, there was little confidence in the HSR due to a perceived lack of transparency in the criteria used to determine the number of stars. Highly processed foods were generally seen as having inflated ratings and participants expressed concern that this would increase consumption of these products. Finally, there was a belief that the HSR had a lack of negative imagery limiting the dissuasive impact on consumers when presented with low-rated foods. Consumers saw benefits in the HSR but were sceptical about how the ratings were derived. Transparency about the development and education on the application may assist with consumers’ perception of the HSR.

## 1. Introduction

Nutrition labelling on food allows consumers to be informed about the nutritional composition of the products they are purchasing [1]. Nutrition labelling on packaging has been demonstrated to improve consumers’ ability to assess product healthiness and encourage healthier food choices [2]. The display of nutrition information on packaged goods is mandatory in many countries [3] and typically comes in the form of a nutrition information panel (NIP) and ingredients list. The NIP displays numerical nutrition information on the side or back of a package, which is not always obvious to the consumer. In contrast, front-of-pack nutrition labels (FoPLs) are more likely to facilitate exposure to nutrition information as they are visible at the moment of choice [4]. 

FoPLs can be categorised according to factual information versus a continuum proposed by Kleef and Dagevos [5]. At one end of this continuum, the ‘purely reductive’ FoPL presents factual information condensed from the NIP and leaves the evaluation up to the consumer. The impact is therefore likely determined by the consumer’s understanding of the facts provided. At the other end of the continuum are the ‘purely evaluative’ FoPLs that are binary in nature because they depict whether a product meets a particular nutrition standard through the presence or absence of the label, typically with a simple graphic such as a tick or stamp. The impact is reliant upon the consumer’s awareness of the meaning of the graphic and the evaluation of the nutrition standard that is being met. 

In the middle of the continuum are ‘hybrid’ FoPLs that present a combination of information from the NIP and an evaluation of that information. A preference for the appearance and use of hybrid compared to purely reductive FoPLs has been found in previous research [2,6,7]. It has been suggested that consumers are generally able to perform tasks related to identifying healthier food products when using a hybrid FoPL compared to a purely reductive version [8,9]. 

In 2014, the Health Star Rating (HSR) [10] was introduced for use on packaged foods in Australia. The hybrid scheme displays an evaluative component based on an algorithm-derived star rating from half a star (least healthy) to five stars (most healthy). Foods high in energy, saturated fat, sodium or total sugar are assigned lower star ratings than similar foods with fewer of these components. The star rating is increased based on the amount of fruits, nuts, vegetables, legumes, and in some cases protein and dietary fibre in the food. The HSR also presents a reductive component, that is, numeric nutrient information per 100 g or 100 mL for energy, sugar, saturated fat and sodium and one additional positive nutrient [10]. The HSR is a voluntary scheme that has recently undergone a formal review after a five-year implementation period. The recommendations from the report suggest some changes should be made to the appearance and calculation of the HSR, but with continued support of the system [11].

The aim of this study was to undertake an in-depth exploration of consumers’ perceptions of the HSR considering the visual layout, nutrient information provided and application to a select number of food products. A secondary aim was to explore how consumers’ use nutrition labelling to inform decisions around the healthiness of packaged food.

## 2. Materials and Methods

This study was underpinned by descriptive phenomenology given that the underlying aim was to explore and describe consumers’ experiences of the HSR. A qualitative approach with focus group discussions was used to gain a rich understanding and comparison of consumers’ perceptions of the HSR. Participants were recruited by the third author (male) using supermarket intercept convenience sampling from two major supermarket chains in regional locations in Queensland, Australia. Two additional participants were recruited through snowball sampling. Participants were considered eligible for the study if they were over 18 years and reported to do at least half of the shopping for themselves or their household. Those with nutrition education were excluded from the study. No prior relationship existed between the researchers and participants. All participants gave their informed consent prior to participation. This study was approved by the Human Research Ethics Committee of the University of the Sunshine Coast (S/14/709).

Four semi-structured focus group discussions involving 15 participants were conducted and moderated by the third author who was previously trained in focus group facilitation. Focus group discussions were guided by an interview protocol designed by the research team based on inquiry logic that was informed by the literature and the study aims (Table 1). A pilot focus group was conducted, feedback sought and the order and wording of some questions were modified accordingly. Participants were asked if they consider the healthiness of food when grocery shopping, and if so, how this is determined. All other questions were delivered following the presentation of props, beginning with four A4 pages (297 × 210 mm) showing different examples of the full HSR format which includes the HSR, and the energy and nutrient icons [12]. This approach invited participants’ initial impression of the visual layout and the nutrient information provided by the HSR outside of the context of a food package. Five pairs of nutritionally equivalent packaged products (breakfast cereal, artificially sweetened carbonated beverage, cordial, crackers and sweet biscuits) that differed in their FoPL scheme were used as food props. First, products labelled with the Daily Intake Guide (%DI) were presented, and this was followed by those labelled with the HSR. The %DI is a reductive scheme that represents the energy or nutrient content per serve as a percentage of a standard reference value [13]. This allowed for comparisons between labelling formats and also facilitated discussion about perceptions of the HSR within the context of food products. The food props were purchased from a major supermarket in regional Australia and were selected based on availability of HSR-labelled products prior to commencement of the first focus group. Focus group discussions lasted between 35 and 65 min and were audio recorded and transcribed with permission from the participants.

Focus group transcripts were thematically analysed by two members of the research team (LS and JR) using an iterative approach as described by Srivastava and Hopwood [14]. Analysis followed the process described by Green et al. [15], where researchers initially immersed themselves in the data, then conceptualised the parts of the data that addressed the research questions into codes. Similar codes were then grouped into broader categories and connections between categories were examined. Categories that emerged were considered important based on length, depth of discussion, order of emergence as well as tendency to appear in more than one focus group. Explanations and interpretations of categories as themes were discussed and agreed upon by the research team in a process of peer debriefing [16] in order to increase the trustworthiness of findings. As all researchers had expertise in nutrition and health, bracketing was employed to further improve the trustworthiness of the data, whereby the researchers attempted to suspend their own perspectives and biases in order to focus on the participants’ descriptions of their experience during the focus groups [17]. Focus group recruitment ceased when no new relevant information emerged.

## 3. Results

Three men and twelve women participated in the focus groups. Nine were over the age of 50, three were aged between 35 and 49 and three were aged between 25 and 34; four participants reported to be educated at a postgraduate level, four at a bachelor level and the remainder had high school, diploma or trade training. Participants’ purchasing behaviours were influenced by various factors including individual health conditions, personal nutritional priorities, allergies, food safety, weight control, taste and price. The NIP was used by most participants to help determine the healthiness of a product, rather than use of any particular FoPL. Sugar, fat, saturated fat and food additives were the nutrients of most interest.

### 3.1. Themes

Three key themes relating to participants’ perceptions of the HSR emerged in the focus group discussions: (1) Practicality of the HSR; (2) Lack of confidence in the HSR; and (3) Lack of dissuasive impact of the HSR, as described below.

#### 3.1.1. Theme One: Practicality of the HSR

Participants’ first impression of the HSR was that it appeared simple, uncluttered and easy to understand. The stars were a commonly recognized symbol that most participants understood to relate to the healthfulness of the product on a scale of half a star to five.

*“I think it’s really straightforward, really obvious, really easy to get a quick glance at something of how many stars it is that’s a really common visual reference for many people of one out of five stars or five out of five stars being good or bad so it’s quite easy to read.”*

*(Participant 9)*

In each of the four focus groups, participants related the HSR to the energy rating used on electrical appliances in Australia [18]. This familiarity made it easier to understand how to utilize the label without guidance.

*“I think the stars are good in that people are already familiar with appliances, so you don’t have to fully educate them on what the concept is.”*

*(Participant 4)*

The explanatory text was also pointed out as being a useful visual aid as it provided context to the numerical information on the HSR. Most participants believed that the HSR would facilitate comparisons between similar products at a glance. In this way, participants felt that the HSR would influence them to purchase higher-rated products.

*“Yeah if I went in and saw my regular chips that I was going to get and they were like 1 star and these ones were right next to them and they’re 3 stars, yeah sure I’d try them.”*

*(Participant 10)*

Most participants felt that higher-rated products still required supporting information and verification that was only available by checking the NIP and ingredients list. Most participants preferred the NIP and the ingredients when making purchasing decisions as they were viewed to be more transparent, thorough and credible sources of nutrition information.

*“I think I’d still look at the table on the back and then make my own assessment from that. There’s no spin or there’s no kind of magic numbers.”*

*(Participant 5)*

The HSR was seen by most participants to be aimed towards people who were time poor.

*“A busy mum with three kids who’s doing a weekly shop isn’t necessarily going to have time to thoroughly read the label.”*

*(Participant 6)*

#### 3.1.2. Theme Two: Lack of Confidence in the HSR

There was a general lack of confidence in the HSR. Many participants viewed there to be a lack of transparency in the process used to determine the ratings. Participants felt there was an incongruence between their perceptions of the healthiness of a food product and the respective star rating. A number of participants were sceptical of the food industry, with concerns that food companies would change the nutritional makeup of their products to increase their HSR. These participants felt that rather than making the food products healthier, the reformulation would be superficial and exploitative.

*“...companies will just manipulate it. They will make subtle changes. Add things, take things out for their product to exploit the algorithm.”*

*(Participant 4)*

Participants were interested in the governing body behind the HSR and the nutrition science used to develop the algorithm. Although most participants trusted the HSR governance, there was suspicion around the evidence base underpinning the algorithm, with some participants voicing concern around the food industries involvement in boosting ratings.

*“The sceptic in me says that those scientists are lobbied by the food industry to present things that will be favourable towards agricultural, you know, whatever.” *

*(Participant 2)*

Other participants felt that consumers needed to be aware of how the algorithm works because an informed consumer is less able to be manipulated.

*“I support any measure that helps people make more informed choices on the nutritional value of food absolutely, I just want to be able to trust that that is, that people understand what’s behind, how these things are rated.” *

*(Participant 5)*

Participants voiced more concern around the ratings of foods towards the lower end of the scale. They felt that ratings seemed to be inflated for some foods they deemed to be ‘junk foods’ with little or no health value. These foods were products with ratings ranging from one and a half to two stars and included the cordial, sweet biscuits and the artificially sweetened carbonated beverages. It was generally the high proportions of sugar in the first two products and the additives in the third that led to dissonance with the ratings.

*“I still think that that rating is very high with all that sugar in it. I’ve got a problem with how they’ve come up with this rating, this number.” *

*(Participant 15)*

Participants appeared to have more confidence in the HSR when rating foods at the higher end of the scale. This view is shown through the following quote relating to the five-star rating of a breakfast cereal.

*“Well five stars is pretty unequivocal, it’s pretty clear. You’re not going to get away with claiming 5 stars unless you can back it up.” *

*(Participant 4)*

#### 3.1.3. Theme Three: Lack of Dissuasive Impact of the HSR

Many participants expressed concerns that the HSR did not appropriately dissuade consumers from purchasing lower-rated products. This view is illustrated in the following quote relating to a product with a rating of two stars:

*“Ok that’s less than 50% so logic would tell you that it’s not so healthy but even so, 2 is sounding reasonable.” *

*(Participant 15)*

It was suggested that low ratings could be accompanied by a symbol reinforcing the negative. Incorporating traffic light colouring into the HSR was also suggested in two of the four focus groups to address this perceived limitation.

*“Even like a colour scheme wouldn’t be a bad thing if that one over there had a green star and this one’s got a red star you’re like “Woah, that’s bad.”” *

*(Participant 10)*

The framing of the label as a ‘Health Star Rating’ was also viewed as potentially confusing, as participants felt that this indicates that there is an absolute health value to any food with a rating.

## 4. Discussion

This study explored consumers’ perceptions of the HSR. Three key themes emerged from the results, namely the practicality of the HSR, the lack of confidence in the HSR and the lack of dissuasive impact of the labelling scheme. The HSR was considered useful for quick comparisons across similar products at a glance due to its summary indicator, which was predicted to be particularly useful in the grocery shopping environment. The nutrient information provided on the HSR was considered to be important and useful to the participants in this study. Participants responded positively to the simplistic visual design of the HSR, contrary to a global study that found the HSR was perceived to have little visual appeal and ‘not stand out’ [19]. Elements that were identified as simple by the participants were as follows: (1) the explanatory text, (2) the uncluttered design, (3) the picture-based interpretation and (4) the familiarity of the stars. A study by Talati et al. [20] found similar results from focus group discussions, with the HSR being preferred over both the %DI and, to a lesser extent, the traffic light system due to the speed with which an evaluation could be made from the summary indicator saving time and effort while shopping. Similarly, simple FoPLs [21] or graphically representative FoPLs [22] have been shown to be liked by consumers and are considered easier to understand than labels with a lot of numbers and words. Many consumers evaluate as little information as possible to make their purchase decisions [23], and although nutrition content may be considered, it is often a lower priority when grocery shopping than factors such as price, taste and food safety [21,24].

Lack of confidence in the HSR was a key theme that emerged from this study. The provision of information on governance was considered essential to our participants. Transparency in the organisation behind the FoPL is known to increase credibility and trust [21], with well-known and trusted organisations being most credible for consumers [25]. The perception by some participants that labelling schemes are not backed by credible organisations may have influenced their confidence in the HSR by association. This finding suggests that consumers need to be educated about the governing body of the HSR as well as how its application onto products is regulated and monitored. The review of the HSR has suggested that there has been improved transparency in the information provided to consumers through the HSR system website, but greater confidence would be apparent if transferred to Food Standards Australia New Zealand [11].

Participants reacted differently to the idea that the implementation of the HSR may influence the food industry in reformulating their products. Some were concerned that reformulation efforts would be superficial and provide limited health benefits to consumers, whereas others felt that these changes would tangibly improve the nutritional profile of food products. Product reformulation of foods following the implementation of an FoPL has previously been successful [26,27]. The implementation of the HSR has the potential of encouraging food companies to reformulate their products by reducing levels of sugar, sodium and saturated fat, and also by increasing nutrients such as dietary fibre. This has been recently demonstrated in the reformulation of children’s packaged foods [28].

Participants in this study also wanted to know how the star ratings were calculated before they could have confidence in the rating. Scepticism of the algorithm was highest when participants were presented with discretionary foods (artificially sweetened carbonated beverage, cordial and sweet biscuits), which they felt were rated too highly. Participants felt that, in these cases, the mechanics of the algorithm may be flawed, which is concurrent with previous studies indicating transparent labelling criteria to instil consumer trust [2,5,21]. This supports the recommendation to better align the HSR with the Australian Dietary Guidelines through changes to the calculator [11]. Consumers use food labels to make judgements about the food product as well as the food supply system behind the product [29]. Health and nutrition content claims on the front of the pack are often viewed by consumers as ‘just an advertising tool’ [30]. This was the case in our study, where participants felt distrust for the HSR, linking this to the food supply system by making judgements about the credibility of the food manufacturer. On the other hand, the NIP and ingredients list on the side or back of the pack (BoP) were viewed by our participants as highly trustworthy sources of information, which may reflect their confidence in how this BoP nutrient information is determined.

The participants in this study felt that the HSR had a limited ability to dissuade consumers to purchase lower-rated products due to its positively framed imagery. All foods eligible for the HSR scheme obtain, at worst, a half star rating, with the only negative communication being the optional explanatory text indicating a ‘high’ level of one of the key nutrients. It was suggested across several of our focus groups that incorporating some negative framing such as red colouring could help to address this. Similarly, traffic light colouring has been proposed to reduce the complexity of numerical information presented on the HSR [20], and interpretive aids such as colour are viewed favourably by consumers [19]. There is preliminary research to suggest that the inclusion of the traffic light colouring would be an effective way of modifying the HSR [31]. However, the recent review of the HSR did not support the use of the traffic light system [11].

While our recruitment strategy and methodology allowed for a rich understanding into consumers’ perceptions of the HSR, it also led to some limitations in our findings. We conducted four focus groups at which point we reached data saturation, whereby no new themes emerged from the data, giving a high indication of the trustworthiness of our findings [32]. Data saturation is the optimal guide for sample size in qualitative research; however, it is known that a sample size of two or three focus groups will likely capture at least 80% of themes on a topic [33]. The qualitative exploratory nature of our study allowed participants to discuss issues around health, nutrition and labelling with high levels of passion, confidence and articulation, which suggests the findings from this study provide authentic insight into consumers’ perception of the HSR, but they may not be generalisable to the wider Australian population. Our participant demographics also showed some variance when compared with the general grocery shopper population in Australia. Eight out of 15 (53%) participants had a bachelor or postgraduate education, compared to approximately 33% of Australian adults in the general population [34]. A further limitation of this study was the small range of HSR-labelled food products that were available for use as food props in the focus groups at the time of this study. An increasing number of products currently display the HSR, and it is recommended that future studies select foods that reflect the full range of products that consumers are exposed to in the supermarket environment.

This study provides an insight into consumers’ perceptions of the HSR. The HSR was perceived as simple and easy to understand, and as being most useful when comparing across similar products at a glance. It was perceived as less useful for analysing single products in isolation and particularly for lower-rated products due to its positively framed communication design. While our results cannot be generalised to the wider Australian grocery shopper population, this sample of participants indicated that there was a lack of confidence in the HSR. This was due to a variety of reasons including a lack of familiarity, a lack of transparency on how the ratings are calculated and a disagreement with the product ratings.

## 5. Conclusions

There is a need to improve consumer confidence in the HSR to ensure accurate guidance when navigating the modern food environment. Campaigns to promote the use of the HSR should focus on improving consumer understanding and the evidence base underpinning the HSR.

## Figures and Tables

**Table 1:** Questions from the focus group questionnaire on the Health Star Rating.

1. When you are purchasing food, do you consider the healthiness of the food product? If so, how do you work out if a food product is healthy? 2. [Show A4 printouts of the HSR] What are your first impressions of the HSR?
3. How do you feel about the look and design of the HSR?
(a) Which parts do you like and why?
(b) Which parts don’t you like and why?
4. How do you feel about the nutrition information presented on the HSR?
(a) What information is useful and why?
(b) What information is not useful and why?
(c) What information do you wish was on there and why?
5. [Shows food products with the %DI label] How do you perceive the healthiness of this product?
6. [Shows food products with the HSR label] How do you perceive the healthiness of these same food products now?
7. How could the HSR be improved? In what ways do you feel this would be an improvement?
8. Is there anything else that you’d like to discuss regarding the HSR?
9. Could you summarise your perceptions around the HSR?
